# Sharing Clinical Notes in Psychotherapy: A New Tool to Strengthen Patient Autonomy

**DOI:** 10.3389/fpsyt.2020.527872

**Published:** 2020-10-28

**Authors:** Charlotte R. Blease, Jan Walker, John Torous, Stephen O'Neill

**Affiliations:** ^1^OpenNotes, General Medicine and Primary Care Research, Beth Israel Deaconess Medical Center and Harvard Medical School, Boston, MA, United States; ^2^Department of Psychiatry, Beth Israel Deaconess Medical Center and Harvard Medical School, Boston, MA, United States

**Keywords:** psychotherapy ethics, open notes, patient autonomy, informed consent, electronic health records, evidence- based practice, psychotherapy

Clinical psychologists and psychotherapists have an ethical duty to respect patient autonomy (1). This means that before a patient can consent or decline to undergo psychotherapy, clinicians are obliged to present adequate and understandable information about the benefits and risks of the treatments. This conceptualization of informed consent differs from the written legal document executed before many medical procedures in the US. Before patients' right to self-determination, providing the appropriate information may also lead to positive therapeutic benefits: demystifying the psychotherapy process can reduce anxiety, enhance patient trust, and strengthen the therapeutic alliance (2). However, informed consent to psychotherapy is “still not routine” (3) and evidence suggests that its importance is, “probably vastly underestimated by many psychologists” (4).

Against the current shortcomings with disclosure practices in psychotherapy, healthcare is becoming more transparent and “open notes” —inviting patients to read their clinical notes via online portals—is a growing movement. Numerous health institutions in over a dozen countries have begun to share health records with patients (5). In the USA, from November 2020, new federal rules mandate, with few exceptions, the sharing of medical notes; psychotherapy notes remain exempt from this ruling (6). Although fewer health organizations have chosen to share mental health notes (7), all patients have the right to understand their care (8). While many clinicians anticipate workflow problems from sharing notes (9, 10), studies suggest that clinicians do not experience major burdens to documentation practices (11–13).

Addressing the perceived challenges with informed consent processes in psychotherapy contexts, we propose that open notes may provide an important new strategy to strengthen patient autonomy and improve clinical outcomes without sacrificing professional autonomy.

## Failures and Perceived Challenges of Informed Consent to Psychotherapy

Surveys across different psychotherapy modalities indicate that many therapists may still fail to disclose relevant information about the nature of the treatment being offered, with practitioners expressing divergent views about the importance of informed consent (14, 15). Psychiatrists and practitioners of psychodynamic psychotherapy appear to be more skeptical about the value of informed consent than adherents of other psychotherapy schools, such as cognitive behavioral therapy (14, 16).

In light of ongoing debates about what constitutes evidence in psychotherapy (17–19), questions about the kind of information that should be disclosed presents a challenge to informed consent. Some psychotherapists may worry about confusing or overwhelming individuals who are already vulnerable or anxious by presenting them with too much information, especially on risks (20). Complicating matters further, consent to psychotherapy is often understood as a process rather than a “one-shot” event with awareness about how psychotherapy works conceived as “procedural knowledge” whereby, it is argued, patients are only able to grasp the nature of therapy as a result of participating in the process (16).

Acknowledgment of these challenges, however, does not obviate the importance of furnishing patients with adequate, relevant, and comprehensible disclosures about psychotherapy (2, 21, 22). Patients have a right to obtain accessible information about the range and nature of psychotherapy treatment options relevant for their condition. Prospective patients should be advised about the estimates of the timing and duration of the treatments, and the risks associated with different psychotherapy options including the decision to decline treatment (23). Although still not standard practice across all psychotherapy traditions, ethicists have strongly urged that brief disclosures about the techniques associated with different modalities should also be communicated (3, 21, 24).

Relatedly, despite disagreements about the relative value of specific techniques in psychotherapy (e.g., of cognitive restructuring in cognitive-behavioral therapies, insight-oriented techniques in psychodynamic therapies) (25–27), there is a widespread agreement among psychotherapy traditions and researchers that the non-specific or so-called “common factors,” such as the working alliance and therapist empathy, mediate the outcomes. Some ethicists propose that there is a moral duty to communicate this to patients (21, 24, 28–30).

Notwithstanding advancements in psychotherapy ethics about the kinds of information that should be disclosed to patients, advice about how best to communicate it remains generic: for example, recent recommendations suggest that disclosures should be conveyed “*verbally and in written form*” [e.g., (21)]. Considerably less attention has focused on how to convey relevant information effectively. Indeed, it is estimated that between 40 and 80% of verbally communicated information in clinical encounters is immediately forgotten or misremembered (31)—a figure that is likely to be even higher among persons who are stressed, anxious, or depressed. Such confusions and misunderstandings may also be exacerbated if patients are reluctant to ask for more information out of embarrassment, fear of “doctor-bothering,” or being perceived as a difficult patient (32).

## Open Notes: A Tool for Patient Autonomy

We propose that giving patients access to their clinical notes may provide an important route to support informed consent in psychotherapy by enhancing patient autonomy, procedural knowledge, and recall about psychotherapy processes.

### Enhancing Relational Autonomy

Many clinicians predict that reading clinical notes might lead to widespread patient confusion (9, 12, 33). However, recent research suggests that when patients are invited to read their mental health notes, this can enhance patient empowerment (7, 34). Expanding on these preliminary findings, we suggest that open notes in mental health contexts may be a valuable tool to augment patient autonomy when this is understood as a relational construct. “Relational autonomy” is the idea that an individual's capacity to make autonomous decisions is socially–situated, and contingent on interpersonal relations and dependencies. By signaling trust in the patient as a “grown-up” care partner, and by facilitating greater time to reflect on disclosures, open notes may strengthen patients' sense of agency and can conceivably play a role in improving outcomes (35). Patients express considerable interest in accessing their clinical records, including their mental health notes, and surveys suggest that only small numbers of patients are confused by what they have read (7, 12, 36, 37). These findings are supported by qualitative research where many patients describe enhanced levels of trust and confidence in clinicians, greater understanding about their health and treatment plans, and feelings of personal validation on reading their notes (7, 38–40).

In interpreting autonomy as a relational concept, the role of patients' trust in clinicians and the strength of the therapeutic alliance are crucial factors to foster a sense of control. Aside from providing adequate and understandable information disclosures, the therapeutic tone and content of clinical notes may also play a causal role in strengthening or diminishing patient autonomy. Importantly, however, some patients do report negative consequences of reading their notes. For example, some survey respondents report being offended or feeling judged by what they read, or detect inconsistencies between the notes and what transpired in therapy sessions (7, 34). Therefore, to cultivate relational autonomy, clinicians will require training on how to write clear, accurate, respectful, and supportive notes (8, 41–43).

### Fostering Procedural Knowledge

As noted, a major perceived difficulty with informed consent to psychotherapy is adequately communicating procedural aspects of care; yet, as a result of accessing their psychotherapy notes some patients report a better understanding of what goes on in sessions and greater insight into their personal goals and their progress (7, 34, 36); for example: “*[H]elps affirm what I'm working on*” (7). Supporting these findings, in a recent study involving patients' access to psychotherapy notes (*n* = 85), more than half of those surveyed reported that reading their notes was “very important” or “extremely important” for feeling in control of their care (7). Similarly, some psychotherapists observe patients as demonstrating a firmer grasp of what goes on in therapy as a result of reading their clinical notes; as one clinician observed: “*[I]t lessens that knowledge gap between the treatment team and the patient in terms of what we're working towards and how…”* (44).

### Improving Patient Recall and Engagement

Although research has not directly explored the use of open notes in communicating information about psychotherapy treatment options, techniques, or information about the common factors, evidence from primary care suggests that rapid online access to clinical notes may help to address the limitations of one-shot disclosures. In major surveys, significant numbers of respondents report better remembering next steps, test results, and referrals (45), and improved adherence to their medications (46).

Preliminary evidence also suggests that patients may derive similar benefits from reading their mental health notes by facilitating recall about what was discussed in psychotherapy sessions; as one patient attested, “*[M]y notes came in handy when I had a really bad breakdown. I walked through all of the steps that she taught me”* (7). Benefits in helping patients with homework and other skills rehearsal are also clear. Recent findings also suggest that access to clinical notes can deepen patient engagement (36); for example: “*Better informed and aware of when something needs clarification”* (7).

### Limitations

Although findings indicate that open notes in the context of psychotherapy presents a promising approach to augment patient autonomy, currently, survey research has been restricted to small sample sizes limiting the generalizability of the results. As with all surveys, findings are based on self-report which may be biased in favor of participants who feel strongly about open notes, or those who are already more engaged with their psychotherapists and/or health care. Many psychotherapists keep very brief notes and, instead of generalized statements around progress toward goals, the full benefit of sharing will only be realized when proper documentation of sessions is shared. Finally, both in psychotherapy and psychiatry settings, further research is required to investigate whether individuals suffering from severe and persistent mental illness are more vulnerable to harm, anxiety, or confusion as a consequence of accessing their notes.

## Conclusions

Informed consent to psychotherapy presents distinctive problems, including perceived barriers. It is the duty of clinicians to find ways to overcome these challenges. Open notes may present a novel solution to extend the patient's visit into “online” settings facilitating disclosure about psychotherapy treatment options, techniques, and other information relevant to decisions about care. To ensure ethical best practice and to harness benefits, and prevent harms (47), we strongly advocate a thorough and practical training and support for psychotherapists on how to write notes that are clear, helpful, and comprehensive, and how to open up dialogue with patients about questions and concerns with what they read in clinical sessions. Such training will not be a “one-shot” event, instead, we envisage that open notes in psychotherapy will require a change of mindset in education and practice. Inevitably, this also mandates a deeper debate about the training of therapists who have learned and adopted styles of note-keeping over the years, or even decades of service. Culture change in healthcare is never easy. However, open notes are here to stay. We argue that clinical notes should not merely be regarded as a repository for documenting patients' health, rather, open notes should be reconceived as a tool with the potential for ethical functionality—one that has the capacity to strengthen patient autonomy.

## Author Contributions

CB wrote the first draft. JW, JT, SO'N, and CB revised the draft multiple times until all authors signed off on the manuscript. All authors contributed to the article and approved the submitted version.

## Conflict of Interest

The authors declare that the research was conducted in the absence of any commercial or financial relationships that could be construed as a potential conflict of interest.
