# Corrigendum: Sharing Clinical Notes in Psychotherapy: A New Tool to Strengthen Patient Autonomy

**DOI:** 10.3389/fpsyt.2020.636411

**Published:** 2021-01-18

**Authors:** Charlotte R. Blease, Jan Walker, John Torous, Stephen O'Neill

**Affiliations:** ^1^OpenNotes, General Medicine and Primary Care Research, Beth Israel Deaconess Medical Center and Harvard Medical School, Boston, MA, United States; ^2^Department of Psychiatry, Beth Israel Deaconess Medical Center and Harvard Medical School, Boston, MA, United States

**Keywords:** psychotherapy ethics, open notes, patient autonomy, informed consent, electronic health records, evidence-based practice, psychotherapy

In the original article, there were errors.

1. A correction has been made to the Introductory section, under main heading of paper,
  Paragraph 2: “Numerous health institutions in over a dozen countries have begun to share health records with patients.”
2. A correction has been made to the section under heading **“FAILURES AND PERCEIVED CHALLENGES OF INFORMED CONSENT TO PSYCHOTHERAPY”**
  Paragraph 1: “such as cognitive behavioral therapy.”
3. A correction has been made to the section under heading: “**FAILURES AND PERCEIVED CHALLENGES OF INFORMED CONSENT TO PSYCHOTHERAPY”**
  Paragraph 2: “worry about confusing or overwhelming individuals.”
4. A correction has been made to the section under heading: “**FAILURES AND PERCEIVED CHALLENGES OF INFORMED CONSENT TO PSYCHOTHERAPY”**
  Paragraph 4: “Some ethicists propose that there is a moral duty to communicate this to patients.”
5. A correction has been made to the section under heading: “**FAILURES AND PERCEIVED CHALLENGES OF INFORMED CONSENT TO PSYCHOTHERAPY”**
  Paragraph 5: “Notwithstanding advancements in psychotherapy ethics about the kinds of information that should be disclosed to patients.”
6. A correction has been made to the section under heading: “**OPEN NOTES: A TOOL FOR PATIENT AUTONOMY”**
  Paragraph 1: “We propose that giving patients access to their clinical notes may provide an important route to support informed consent in psychotherapy by enhancing patient autonomy.”
7. A correction has been made to the section under heading: “**OPEN NOTES: A TOOL FOR PATIENT AUTONOMY** and subheading **Enhancing Relational Autonomy”**
  Paragraph 1: “this can enhance patient empowerment.”
8. A correction has been made to the section under heading: “**OPEN NOTES: A TOOL FOR PATIENT AUTONOMY** and subheading **Enhancing Relational Autonomy”**
  Paragraph 1: “These findings are supported by qualitative research where many patients describe enhanced levels of trust.”
9. A correction has been made to the section under heading: “**OPEN NOTES: A TOOL FOR PATIENT AUTONOMY** and subheading **Enhancing Relational Autonomy”**
  Paragraph 2: “relational concept, the role of patients' trust in.”
10. A correction has been made to the section under heading: “**OPEN NOTES: A TOOL FOR PATIENT AUTONOMY** and subheading **Improving Patient Recall and Engagement”**
  Paragraph 1: “suggests that rapid online access.”
11. A correction has been made to the section under heading: “**OPEN NOTES: A TOOL FOR PATIENT AUTONOMY** and subheading **Improving Patient Recall and Engagement”**
  Paragraph 2: “that access to clinical notes can deepen patient engagement.”
12. A correction has been made to the section under heading: “**CONCLUSIONS”**
  Paragraph 1: “including perceived barriers.”
13. A correction has been made to the section under heading: “**CONCLUSIONS”**
  Paragraph 1: “and how to open up dialogue with patients.”

The authors apologize for this error and state that this does not change the scientific conclusions of the article in any way. The original article has been updated.

