# Supplementary material for: Sharing Clinical Notes in Psychotherapy: A New Tool to Strengthen Patient Autonomy
Source: Front Psychiatry. 2020 Oct 28;11:527872. doi: 10.3389/fpsyt.2020.527872 (PMC7655789; doi:10.3389/fpsyt.2020.527872)
Supplement: Supplementary file 1 [file Data_Sheet_1.PDF]

Box 1: Key Questions and Findings

What is already known about this topic?

- Informed consent to psychotherapy is still not standard practice.
- Perceived challenges include: issues about what should be disclosed, risks of overwhelming patients by providing too much information, and the conception that knowledge of psychotherapy is best obtained by undergoing the process.
- Open notes is an international movement in healthcare with the goal of providing patients with fully transparent online access to their clinical notes.
- In the context of mental health, preliminary studies indicate that patients experience many benefits and few harms from reading their clinical notes.

What are the new findings?

- Open notes may provide an innovative tool in psychotherapy ethics, facilitating informed consent processes.
- Inviting patients to read their notes may strengthen agentic skills, allow patients greater time to reflect on disclosures, enhance procedural knowledge, and improve engagement and recall about what goes on in psychotherapy sessions.
- To avail of the potential of open notes to enhance ethical psychotherapy practice, clinicians will require education and training in how to write notes that strengthen disclosure processes and foster patient autonomy.
